# Identifying the most at-risk age-group and longitudinal trends of drug allergy labeling amongst 7.3 million individuals in Hong Kong

**DOI:** 10.1186/s12916-024-03250-0

**Published:** 2024-01-26

**Authors:** Valerie Chiang, Andy Ka Chun Kan, Chinmoy Saha, Elaine Y. L. Au, Philip H. Li

**Affiliations:** 1 Division of Clinical Immunology, Department of Pathology, Queen Mary Hospital, Pokfulam, Hong Kong; 2 Division of Rheumatology and Clinical Immunology, Department of Medicine, Queen Mary Hospital, The University of Hong Kong, 102 Pokfulam Road, Pokfulam, Hong Kong

**Keywords:** Allergy, Big data, Drug, Epidemiology

## Abstract

**Background:**

Incorrect drug ‘allergy’ labels remain a global public health concern. Identifying regional trends of drug allergy labeling can guide appropriate public health interventions, but longitudinal or population drug allergy studies remain scarce. We analysed the serial epidemiology of drug allergy labeling to identify specific subgroups at highest risk of drug allergy labeling for potential interventions.

**Methods:**

Longitudinal, population-wide drug allergy labels and clinical data from over 7,337,778 individuals in Hong Kong between 2016 and 2021 were analysed.

**Results:**

The absolute prevalence and incidence of documented drug allergy were 5.61% and 277/100,000 population, respectively. Annual incidence of new allergy labels was stable between 2016 and 2019, until a significant drop in 2020 (−16.3%) during the COVID19 pandemic. The most common allergy labels were anti-infectives (245,832 [44.5%]), non-steroidal anti-inflammatory (106,843 [19.3%]), and nervous system drugs (45,802 [8.3%]). The most common labeled culprits for the most severe immediate-type (anaphylaxis) and non-immediate-type (Stevens-Johnson syndrome) reactions were beta-lactams and nervous system drugs, respectively. For individuals at highest risk of labeling, there was significantly higher incidence of overall drug and beta-lactam allergy labeling amongst individuals aged > 40 years which contributed to the majority of newly labeled allergies (377,004, 68.2%).

**Conclusions:**

Contrary to traditional dogma, we identified disproportionately higher incidence of drug allergy labeling amongst older individuals, rather than the paediatric age group. We advocate for more population-wide drug allergy studies to investigate this phenomenon in other cohorts as well as future preventative and delabeling efforts focusing on the adult population.

**Supplementary Information:**

The online version contains supplementary material available at 10.1186/s12916-024-03250-0.

## Background

Population-based studies have demonstrated the potential of use of big data in clinical and drug allergy research [1–4]. The availability of longitudinal datasets can explore new dimensions of drug allergy research which would not have been possible with only traditional single-centre or cross-sectional studies. To illustrate, we previously reported the largest drug allergy epidemiology study by taking advantage of Hong Kong’s unified electronic healthcare record system, generating and analysing data from more than 95% (over 7 million individuals) of the population [1]. Based on population-wide data, we were able to accurately report the absolute prevalence and annual incidence of documented drug and beta-lactam allergies. However, at the time, we were only able to provide a condensed one-year snapshot on the landscape of drug/beta-lactam allergy and did not further analyse the granularity of individual patient data—such as specific-drug allergy culprits, detailed demographics (such as age at drug allergy labeling) or duration of drug allergy labels. As of writing, there have not been further population-based drug allergy studies in our region.

Incorrect drug ‘allergy’ labels remain a significant public health concern. This is exemplified by the immense burden of misdiagnosed beta-lactam allergy, where up to 90% of labels have been found to be incorrect after allergy evaluation [5]. Inappropriate antimicrobial usage due to of mislabeled beta-lactam allergy is associated with a myriad of adverse clinical outcomes, increased healthcare costs, and development of antibiotic resistance [6]. Preventing or ‘delabeling’ (removing) misdiagnosed drug allergies has therefore been a priority in pharmaceutical stewardship and drug allergy research. However, delabeling efforts have often been limited by the paucity of allergy services and specialists. Analysis of objective and pragmatic parameters from big data research can potentially elucidate longitudinal and regional labeling patterns (which may differ vastly across time and between ethnicities) to help inform future interventions—including targets of public health initiatives and focus of education efforts as well as interventions to prevent incorrect labeling and optimise the utilisation of scarce allergy resources [6, 7].

This study aims to capture the recent 5-year trend of drug allergy labeling in Hong Kong utilising longitudinal population-wide data. We report the serial incidence of drug allergy labeling, detailed analysis of drug allergy labels (including the culprits of the most severe drug allergy reactions), and identify subgroups at higher risk of carrying drug allergy labels.

## Methods

### Collection of anonymised data

Anonymised data from the Hospital Authority was collected by the Information Technology and Health Informatics Division, Hospital Authority Head Office. Informed consent was waived (because all data were anonymous and collected retrospectively), and data extraction was approved by the institutional review board of the University of Hong Kong and Hospital Authority Hong Kong West Cluster (UW 18-669). Hong Kong is a unique entity where the Hospital Authority is the sole publicly funded healthcare system for the entire territory. It possesses facilities in seven regions (Hong Kong East, Hong Kong West, Kowloon Central, Kowloon East, Kowloon West, New Territories East, and New Territories West), comprising 43 hospitals, 49 specialist outpatient clinics, and 73 general outpatient clinics; it provides approximately 90% of all in-patient services [8]. All clinical services utilise a unified ‘Electronic Patient Records’ system, providing medical records for over 7.3 million individuals. This system allows complete and uniform drug allergy data to be gathered and analysed. A snapshot of cross-sectional data from all available medical records was retrieved as of 23 July, 2021. Physician-documented drug allergy labels, age at the time of documented drug allergy, and names of suspected culprits were collected. Furthermore, the annual incidence (from 1 January to 31 December of each year) of newly labeled drug allergy as well as all allergy labels with manifestation documented as ‘anaphylaxis’ and ‘Stevens-Johnson syndrome’ from 1 January 2016 to 31 December 2020 were reviewed. Drugs were categorised according to the British National Formulary Drug Classes, except for non-steroidal anti-inflammatory drugs (NSAIDs), which were separated into its own category [9]. Drugs which were entered as ‘free text’ into the electronic system were checked and converted back to structured drug items when possible. Those ‘free text’ reported drug allergy which could not converted or were incomplete were excluded from analysis. Drugs which did not fit into any drug classes were categorised as ‘Others’ and listed in Additional file 1: Text S1.

### Statistical analysis

All statistics were analysed using IBM SPSS Statistics 27.0 (IBM Corp., Armonk, NY, USA). Prevalence of drug allergy labeling was calculated. Values were presented as numbers (percentages) as appropriate. Age distributions of individuals with and without drug allergy labels were analysed. Trends of incidence of labelled drug allergy were analysed using negative binomial testing. Two-sided *p*-values < 0.05 were considered statistically significant.

## Results

Anonymised data was collected from the electronic health records of 7,337,778 unique individuals. Amongst them, 411,885 had at least one physician-documented drug allergy label in 2021. The absolute prevalence of physician-labeled drug allergy in Hong Kong was therefore 5.61%. A total of 552,897 drug allergy labels were shared amongst the 441,885 unique individuals; 346,969 (62.8%) of the documented allergy labels were found amongst female individuals. Amongst individuals with labeled drug allergy, 28,467 (6.91%) had more than two concomitant drug allergy labels.

**Fig. 1:**
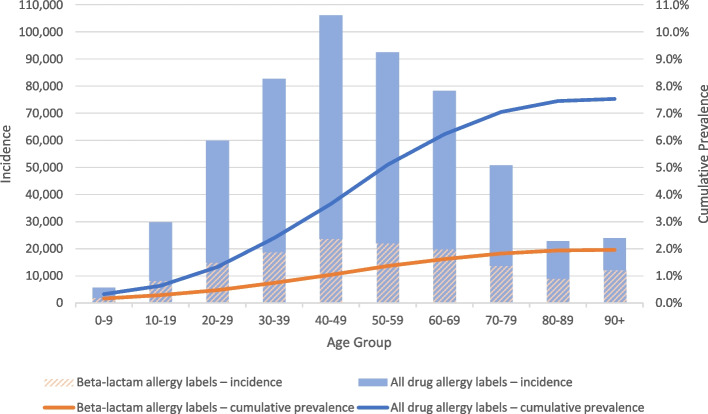
Incidence and cumulative prevalence of drug and beta-lactam allergy labels across age during the study period

The incidence and cumulative prevalence of drug and beta-lactam allergy labels by age group are shown in Fig. 1, and the age distribution of incident drug allergy labeling (per label) in comparison to non-allergic individuals (i.e. those without any reported drug allergy labels) is shown in Table 1. Most drug allergies were labeled at 50–59 years of age (106,249, 19.2%), and more than half of all drug allergies (377,004, 68.2%) were labeled at or above the age of 40.

**Table 1:** Age distribution of incident drug allergy labeling (per allergy label) in comparison to individuals without drug allergy

**Age group (years)**	**Individuals without drug allergy**	**Incident drug allergy labeling**^a^
Overall	6,922,941	552,897
0–9	1,016,452 (14.7%)	23,899 (4.3%)
10–19	640,551 (9.3%)	22,756 (4.1%)
20–29	931,646 (13.5%)	50,844 (9.2%)
30–39	1,061,763 (15.3%)	78,316 (14.2%)
40–49	1,074,751 (15.5%)	92,573 (16.7%)
50–59	963,259 (13.9%)	106,249 (19.2%)
60–69	597,246 (8.6%)	82,814 (15.0%)
70–79	416,292 (6.0%)	59,968 (10.9%)
80–89	186,387 (2.7%)	29,804 (5.4%)
90+	34,594 (0.5%)	5,674 (1.0%)

^a^Some patients may have multiple drug allergy labels

**Fig. 2:**
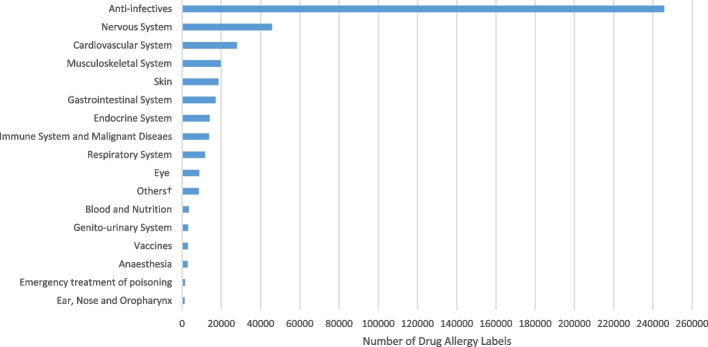
Distribution of all 552,897 drug allergy labels according to drug category. ‘†’ indicates “Others”: please refer to Additional file 1: Text S1

Out of the 552,897 documented drug allergy labels, the most common implicated drugs were anti-infectives (245,832 [44.5%]) and NSAIDs (106,843 [19.3%]), followed by nervous system drugs (45,802 [8.3%]) and cardiovascular system drugs (27,977 [5.1%]) (Fig. 2). Amongst the 245,832 documented anti-infective allergy labels, 143,925 (58.5%) were towards beta-lactams (Table 2). Amongst beta-lactams, penicillins accounted for the vast majority (119,274 [82.9%]) of reported culprits. Amoxicillin-clavulanate was the most commonly labeled (30,396 [21.1%]), followed by phenoxymethylpenicillin (penicillin V) (25,678 [17.8%]), amoxicillin (21,806 [15.2%]), ampicillin (16,335 [11.3%]), and cloxacillin (9,689 [6.7%]).

**Table 2:** Distribution of reported allergy labels toward anti-infectives

Total number of drug allergy labels	245,832
**Drug class/name**	
Beta-lactams, *n* (%)	143,925 (58.5)
Tetracyclines, *n* (%)	22,711 (9.24)
Anti-fungals, *n* (%)	17,797 (7.2)
Macrolides, *n* (%)	17,301 (7.0)
Quinolones, *n* (%)	16,844 (6.9)
Nitroimidazole, *n* (%)	5975 (2.4)
Amphenicols, *n* (%)	4670 (1.9)
Aminoglycosides, *n* (%)	3803 (1.5)
Glycopeptides, *n* (%)	2591 (1.1)
Other anti-infectives, *n* (%)	2529 (1.0)
Anti-virals, *n* (%)	2107 (0.9)
Anti-mycobacterial, *n* (%)	1928 (0.8)
Lincosamides, *n* (%)	1751 (0.7)
Anti-protozoals, *n* (%)	1287 (0.5)
Sulphonamides, *n* (%)	531 (0.3)
Anti-helminths, *n* (%)	82 (0.0)

Thereafter, we analysed drug allergy labels specifically with reported manifestations of ‘anaphylaxis’ and ‘Stevens-Johnson syndrome’. Between 2016 and 2020, there were a total of 1325 reported cases of drug-induced anaphylaxis (0.32% of drug allergy) and 1706 cases (0.41% of drug allergy) of Stevens-Johnson syndrome. Breakdown of labeled drug culprits are shown in Tables 3 and 4, respectively. Anti-infectives (544 [41.1%]) were the most commonly labeled culprits of drug-induced anaphylaxis (in particular, beta-lactams: 402 [30.3%]), followed by musculoskeletal system drugs (158 [11.9%]) and nervous system drugs (157 [11.8%]). Anti-infectives (735 [43.1%]) were also the most common culprits of Stevens-Johnson syndrome (in particular, beta-lactams 348 [20.4%]), followed by nervous system drugs (390 [22.9%]) and musculoskeletal system drugs (348 [20.4%]).

**Table 3:** Reported culprits of drug-induced anaphylaxis according to drug category

Total number of patients with reported drug-induced anaphylaxis	1325
**Drug class/name**	
Anti-infectives, *n* (%)	544 (41.1)
Beta-lactams, *n* (%)	402 (30.3)
Quinolones, *n* (%)	30 (2.3)
Macrolides, *n* (%)	25 (1.9)
Aminoglycosides, *n* (%)	24 (1.8)
Glycopeptides, *n* (%)	19 (1.4)
Anti-protozoals, *n* (%)	13 (1.0)
Sulphonamides, *n* (%)	9 (0.7)
Tetracyclines, *n* (%)	9 (0.7)
Anti-fungals, *n* (%)	4 (0.3)
Lincosamides, *n* (%)	3 (0.2)
Anti-virals, *n* (%)	2 (0.2)
Anti-mycobacterials, *n* (%)	1 (0.1)
Other anti-infectives, *n* (%)	3 (0.2)
Musculoskeletal system, *n* (%)	158 (11.9)
Nervous system, *n* (%)	157 (11.8)
Anaesthesia, *n* (%)	155 (11.7)
Immune system and malignant diseases, *n* (%)	99 (7.5)
Gastrointestinal system, *n* (%)	46 (3.5)
Respiratory system, *n* (%)	28 (2.1)
Blood and nutrition, *n* (%)	25 (1.9)
Skin, *n* (%)	19 (1.4)
Cardiovascular system, *n* (%)	19 (1.4)
Eye, *n* (%)	12 (0.9)
Endocrine system, *n* (%)	10 (0.8)
Ear, nose and throat, *n* (%)	6 (0.5)
Vaccines, *n* (%)	4 (0.3)
Genito-urinary system, *n* (%)	1 (0.1)
Emergency treatment of poisoning, *n* (%)	1 (0.1)
Unknown drug culprit, *n* (%)	40 (3.0)

**Table 4:** Reported culprits of Stevens-Johnson syndrome according to drug category

Total number of patients with reported Stevens-Johnson syndrome	1706
**Drug class/name**	
Anti-infectives, *n* (%)	735 (43.1)
Beta-lactams, *n* (%)	348 (20.4)
Sulphonamides, *n* (%)	81 (4.7)
Tetracyclines, *n* (%)	70 (4.1)
Quinolones, *n* (%)	64 (3.8)
Macrolides, *n* (%)	50 (2.9)
Anti-mycobacterials, *n* (%)	31 (1.8)
Anti-protozoals, *n* (%)	22 (1.3)
Aminoglycosides, *n* (%)	21 (1.2)
Anti-virals, *n* (%)	14 (0.8)
Glycopeptides, *n* (%)	12 (0.7)
Anti-fungals, *n* (%)	8 (0.5)
Lincosamides, *n* (%)	7 (0.4)
Other anti-infectives, *n* (%)	7 (0.4)
Nervous system, *n* (%)	390 (22.9)
Musculoskeletal system, *n* (%)	348 (20.4)
Cardiovascular system, *n* (%)	55 (3.2)
Gastrointestinal system, *n* (%)	39 (2.3)
Respiratory system, *n* (%)	33 (1.9)
Anaesthesia, *n* (%)	24 (1.4)
Immune system and malignant diseases, *n* (%)	16 (0.9)
Ear, nose and throat, *n* (%)	8 (0.5)
Eye, *n* (%)	7 (0.4)
Skin, *n* (%)	5 (0.3)
Blood and nutrition, *n* (%)	5 (0.3)
Genito-urinary system, *n* (%)	4 (0.2)
Vaccines, *n* (%)	2 (0.1)
Endocrine system, *n* (%)	2 (0.1)
Unknown drug culprit, *n* (%)	33 (1.9)

**Fig. 3:**
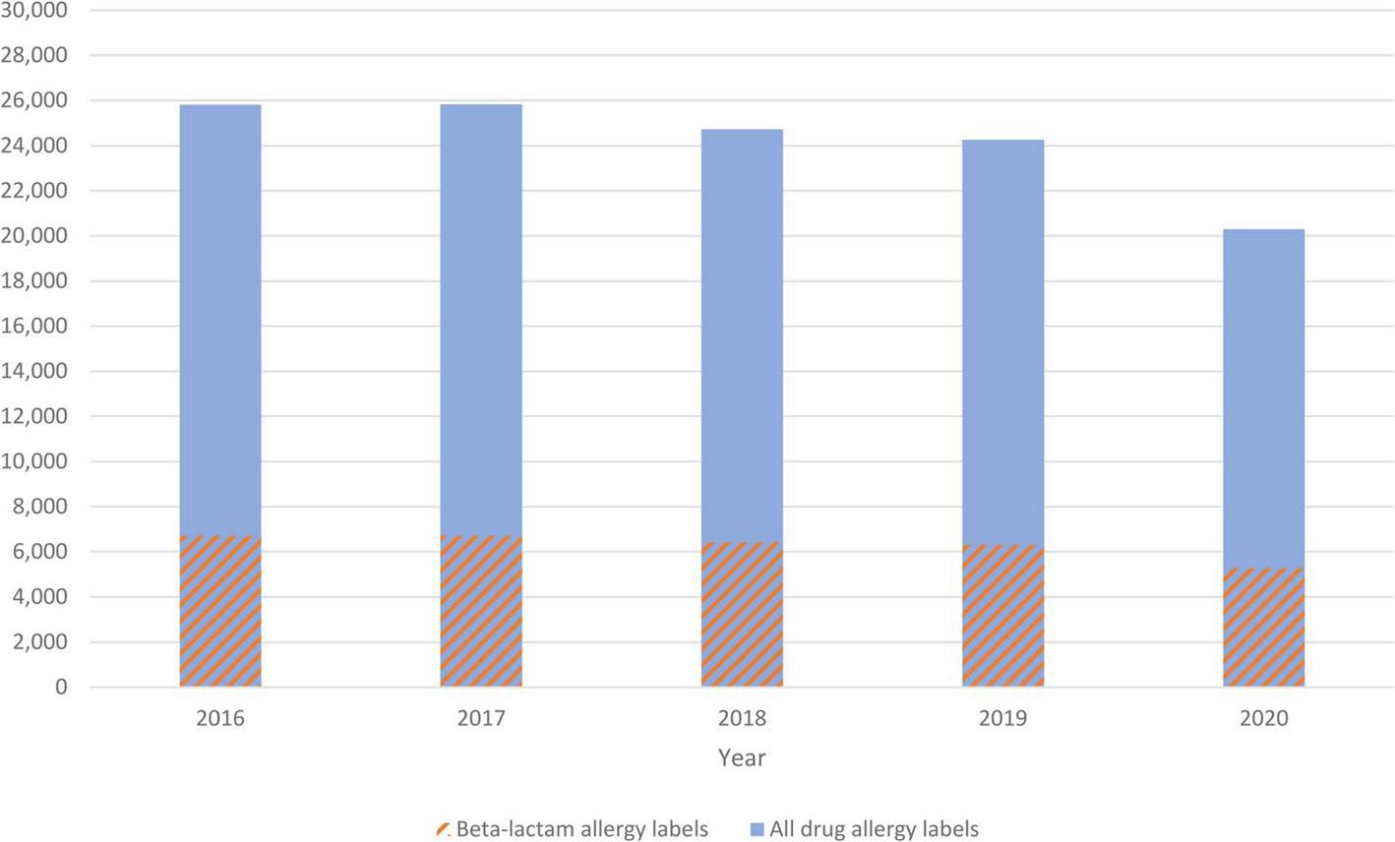
Annual incidence of new drug and beta-lactam allergy labels from 2016 to 2020

In 2016, 25,810 new physician-documented drug allergy labels were created with an incidence of 352 per 100,000 population. The annual incidence remained relatively stable from 2016 to 2017 (352 per 100,000), 2018 (337 per 100,000), and 2019 (331 per 100,000) (*p* = 0.200). There was no significant change in incidence until a significant decrease in 2020 to 277 per 100,000 (*p* = 0.037). The annual incidence and relative differences of physician-documented drug allergy labels are shown in Fig. 3.

## Discussion

Population-wide studies utilising the power of ‘big data’ remains scarce likely due to difficulties in obtaining such data. Emerging and innovative technologies are increasingly used under pharmacovigilance to document adverse drug reactions in general, but with little focus on drug allergy specifically [10–12]. Using our population-wide data, we were able to generate a registry of unprecedented size, comprising more than 7 million individuals, allowing detailed characterisation of the absolute prevalence and incidence and individual drug culprits as well as the age of incident drug allergy labels of our entire population. To the best of our knowledge, this study is the first to investigate the longitudinal incidence of documented drug allergy and labeling patterns on a population-wide basis.

Our study revealed about 1 in 18 (5.61%) of the population has been labeled with a drug allergy, which is substantially lower than prevalence reported by other regions, as well as our own previous hospital-based studies, which range from 20% up to one third [13–18]. This contrast may be attributed to allergy labels that are physician documented, rather than patient-reported or survey-based. It also highlights the impact of population-wide data, as opposed to limited data from electronic health records from large centres, which may restrict the studied population to particular tertiary referral populations, which can be prone to type I errors from inadequate sampling. It is postulated that these individuals may have more medication exposure and are not representative of broader populations [2].

Interestingly, we identified that most drug allergies in Hong Kong were labeled amongst older individuals—with disproportionately higher incidence amongst those above 40 years of age. This is contrary to the traditional dogma reflected from the previous experience from Western cohorts, suggesting the majority of individuals acquire drug allergy labels during childhood and carry them into adulthood [19]. We postulate this stark contrast may be due to region-specific or inter-population diversities, such as in differences in culture, prescribing practices or medication use, rather than genuine biological differences. For example, more than 30% of Hong Kong Chinese report self-medicating (either Western and Chinese medicines, without prescription), and almost 40% of patients report ‘doctor shopping’ (i.e. changing of doctors without professional referral in the same illness) without consulting a regular doctor or family physician [20, 21]. A proportion of patients also prefer to consult Traditional Chinese Medicine practitioners (rather than Western doctors) especially for episodic or acute illnesses [22]. Hence, individuals in Hong Kong may not be as frequently exposed to medications or attend follow-up with their medical physicians to report suspected drug allergies. Any suspected drug allergy reactions may therefore occur later in life or not be documented by their physicians until adulthood.

Alternatively, our findings may also represent a paradigm shift in drug allergy labeling patterns that has remained unnoticed due to the paucity of prior systematic or large population-based studies. Although increasing age has been noted to correlate with allergy label prevalence, likely due to more antibiotic usage over the years, our data reveals that the incidence of allergy documentation peaks around middle age. This highlights the urgent need for future inter-population big data allergy research. Nonetheless, given the disproportionate burden of drug allergy labeling amongst adults in our region, we advocate for more interventions focused towards older individuals. This includes channelling more resources towards training of adult allergists (as there remains significantly fewer adult than paediatric allergists in Hong Kong) and enhance delabeling initiatives as well as educating and empowering frontline healthcare professionals to discern symptoms of genuine allergy and prevent incorrect allergy labeling—especially amongst adult patients [23].

This study also confirms the severity of a beta-lactam/penicillin-dominated drug allergy landscape in Hong Kong and internationally [1–3, 7]. Within anti-infectives, beta-lactams constituted the majority of all documented drug allergy, with penicillins contributing to almost 82.9% of all beta-lactam labels. Beta-lactams were amongst the most commonly reported culprits of drug-induced anaphylaxis and second in Stevens-Johnson syndrome—the most severe immediate- and non-immediate drug allergy reactions, respectively. However, our previous studies revealed that only 10–13.8% of documented penicillin allergy is found to be correct after evaluation [1, 24]. Incorrect labels lead to unnecessary penicillin avoidance, posing immense challenges in antimicrobial stewardship. This is especially relevant in Hong Kong, where there has been an upsurge of various multi-drug resistant organisms [25, 26]. Penicillin allergy labels have also shown to affect geriatric and immunocompromised patients, associated with a multitude of adverse clinical outcomes, including increased healthcare costs, more frequent and longer hospital stays, and even death [14, 27–29].

Overall, anti-infectives were the most commonly reported culprit in the most severe immediate-type (anaphylaxis) and delayed-type (Stevens-Johnson syndrome) hypersensitivity reactions; they comprise 1325 of reported cases of drug-induced anaphylaxis and 1706 cases of Stevens-Johnson syndrome. Nation-wide studies on anaphylaxis performed by other countries also exhibited similar trends of beta-lactams being the most reported culprit drugs [30, 31]. However, iodinated contrast media were most attributed to anaphylaxis in a large Korean study, followed by cefaclor [32]. This is a surprising distinction given the proximity of the region and closer shared cultural ancestry to the Hong Kong population and warrants further study.

Stevens-Johnson syndrome is well-known to exhibit differences between ethnic groups, with implications in associated HLA risk alleles. Limited population-wide studies on Stevens-Johnson syndrome typically identifies allopurinol and anti-epileptic drugs as the most frequent causes [33, 34]. This is similar to findings from smaller reports from the general Asia-Pacific region, in which antiepileptic drugs (carbamazepine, phenytoin, lamotrigine), allopurinol, and antimicrobials are most implicated drugs in severe cutaneous adverse reactions [7, 35–39]. Our study also echoes these findings, but with beta-lactam antibiotics taking up a near-equivalent proportion with nervous system and musculoskeletal system drugs (20.4% vs 22.9 and 20.4%, respectively). The predominance of beta-lactam antibiotics may be due to differences in local prescribing or labeling practices. As these are allergy labels that have not been subsequently confirmed, local doctors may be more inclined to label beta-lactam antibiotics along with anti-epileptics if any patient was exposed to both prior to the onset of symptoms. Presence of multiple labels and prescription histories should be further studied to investigate these hypotheses.

Incidence of drug allergy labels has remained relatively stable over the 5-year study period (331–352 per 100,000 population), until a significant 16.3% drop in 2020 (*p* = 0.029) (Additional file 2: Table S1). This was likely due to the coronavirus disease 2019 (COVID19) pandemic resulting in a population-wide reduction in medical visits, drug prescriptions, or allergy reporting [40]. It would be interesting to see if the incidence of drug allergy labeling will rebound back to pre-COVID19 figures following relaxation of social restrictions. These changes in incidence contrast with previous studies, which found declining trends in both antibiotics and NSAIDs [2].

Despite the severe lack of allergists in the Asia-Pacific region, previous multi-disciplinary initiatives have shown much promise in tackling drug allergies [24, 41, 42]. For example, a nurse-led penicillin allergy delabeling initiative demonstrated superior outcomes compared to traditional allergist evaluation in Hong Kong and similar strategies employing trained pharmacists are underway [24]. Further research on wider applications of similar multidisciplinary drug allergy initiatives is warranted.

Given its observational nature, this study has several limitations. First, this study only focuses on drug allergy labels rather than confirmed genuine allergies. Similarly, although the reported manifestations of the drug allergy label were physician-documented diagnoses, we were unable to clarify if all diagnoses met diagnostic criteria. Second, only limited clinical data was available, and more detailed data such as comorbidities, medication use, or hospitalisation records were not available for further analysis. Third, we focused specifically on the most severe immediate-type (anaphylaxis) and non-immediate-type (Stevens-Johnson syndrome) reactions only, and more detailed analysis of other manifestations are underway. Finally, we did not include reported drug allergies which were entered as ‘free text’ into the electronic health records that could not be manually converted into structured item (often due to incomplete information). These limitations highlight the importance of dedicated and interventional studies in the future.

## Conclusion

In conclusion, population-specific drug allergy analysis can unlock new dimensions of drug allergy research—such as detailed labeling patterns and comparative age distribution of documented drug allergies. We report the longitudinal incidence of documented drug allergy and specific allergy labels as well as identifying disproportionate drug allergy labeling amongst older individuals in our region. Importance of region-specific data is highlighted and can also inform the directions of appropriate public health initiatives in an evidence-based manner. Future inter-regional and inter-ethnic big data studies will be required to confirm external validity of our findings to other populations.

### Supplementary Information

**Additional file 1: Text S1.** Drugs which did not fit into any drug classes and categorized as “Others”.

**Additional file 2: Table S1.** Analysis of the annual incidence of drug allergy labels from 2016 to 2020 using negative binomial regression.

## Data Availability

The datasets generated and/or analysed during the current study are not publicly available due privacy/ethical restrictions but are available from the corresponding author on reasonable request.

## Abbreviations

COVID19: Coronavirus disease 2019
NSAIDs: Non-steroidal anti-inflammatory drugs
SJS: Stevens-Johnson syndrome
