# Supplementary material for: Identifying the most at-risk age-group and longitudinal trends of drug allergy labeling amongst 7.3 million individuals in Hong Kong
Source: BMC Med. 2024 Jan 26;22:30. doi: 10.1186/s12916-024-03250-0 (PMC10811878; doi:10.1186/s12916-024-03250-0)
Supplement: Supplementary file 1 — Additional file 1: Text S1. Drugs which did not fit into any drug classes and categorized as “Others”. [file 12916_2024_3250_MOESM1_ESM.docx]

**Text S1 - Drugs which did not fit into any drug classes and categorized as “Others”:**

ALUMINIUM CHLORIDE HEXAHYRATE

ALUMINIUM POTASSIUM SULPHATE

AMINOGLUTETHIMIDE

AMMONIA + IPECACUANHA + SODIUM CITRATE

AMMONIA AND LIQUORICE

AQUEOUS ARGININE

BARIUM SULPHATE

BENEDICTS SOLUTION

BENZOIN COMPOUND

BISMUTH SUBNITRATE & IODOFORM

BISMUTH SUBNITRATE + IODOFORM + LIQUID PARAFFIN

CALCIUM CARBIMIDE

CASTBLAST

CASTELLANIS

CLOSTRIDIOPEPTIDASE A

COPPER CHLORIDE

CROTAMITON 10%+HYDROCORTISONE 0.25%

DIMETHYLPOLYSILOXANE

ENBUCRILATE TISSUE ADHESIVE

EPIZON RECTOCAP

ETHER SPIRIT

FLUORESCEIN

FLUORODEOXYGLUCOSE F18

FORMALIN GADOBENATE

DIMEGLUMINE GADOBUTROL

GADODIAMIDE GADOXETIC ACID GALLIUM-67 CITRATE

GASTROGRAFIN

GELAFUNDIN

HEPATOFALK

HISTAMINE ACID PHOSPHATE

HYALURONIDASE

HYDROXYETHYL STARCH 6% IN NORMAL SALINE

INDIGO CARMINE

INDOCYANINE GREEN

IOBITRIDOL IODINE AQUEOUS

IODIXANOL

IOHEXOL

IOMEPROL

IOPAMIDOL

IOPODATE SODIUM

IOPROMIDE

IOTHALAMATE

MEGLUMINE

IOTHALAMATE SODIUM

IRIDIUM

HAIRPIN K Y

LIOTHYRONINE

LIPIODOL ULTRA FLUID

LIQUID PARAFFIN 50% + PHENOLPHTHALEIN 0.3%

MAGNEVIST

MALAMIL D

MASTU S

MEGLUMINE GADOTERATE

MEGLUMINE IOTHALAMATE

MEGLUMINE IOXITALAMATE

MENTHOL + EUCALYTUS

METACRESOLSULPHONIC ACID FORMALDEHYDE

METHACHOLINE

METYRAPONE

OMNIPAQUE 300

PANCEBRIN

PAPAIN

PATENT BLUE V

PECTIN

PHENOL LIQUIFIED SWABS

PHENYLALANINE

POLYACRYLIC ACID

POTASSIUM CHLORIDE IN WATER FOR INJECTION

POTASSIUM PERCHLORATE RADIOPAQUE

POLYVINYL CHLORIDE

SILICONE OIL 1300

SODIUM BENZOATE

SODIUM MEGLUMINE IOXAGLATE

SODIUM METABISULPHITE

STANNOUS AGENT KIT

STRONTIUM-89 CHLORIDE

SUCCIMER

SULPHOBROMOPHTHALEIN

SULPHUR HEXAFLUORIDE

SYRUP TALC

TECHNETIUM MEDRONATE

TECHNETIUM MERTIATIDE

TECHNETIUM PYP PREPARATION KIT (TECHNE)

TECHNETIUM SESTAMIBI

TECHNETIUM SODIUM PERTECHNETATE

TECHNETIUM

TETROFOSMIN

THALLOUS CHLORIDE

TROMETAMOL

TURPENTINE

UREA C-13

UROGRAFIN
