# Supplementary material for: Identifying the most at-risk age-group and longitudinal trends of drug allergy labeling amongst 7.3 million individuals in Hong Kong
Source: BMC Med. 2024 Jan 26;22:30. doi: 10.1186/s12916-024-03250-0 (PMC10811878; doi:10.1186/s12916-024-03250-0)
Supplement: Supplementary file 2 — Additional file 2: Table S1. Analysis of the annual incidence of drug allergy labels from 2016 to 2020 using negative binomial regression. [file 12916_2024_3250_MOESM2_ESM.docx]

Table S1. Analysis of the annual incidence of drug allergy labels from 2016 to 2020 using negative binomial regression

| **Year** | **Total number of new drug allergy labels** | **Incidence rate ratio (95% CI) †** | **p-value** |
| --- | --- | --- | --- |
| 2016 | 25,810 | Referent |  |
| 2017 | 25,823 | 1.00 (0.81-1.24) | 0.996 |
| 2018 | 24,718 | 0.96 (0.77-1.19) | 0.694 |
| 2019 | 24,268 | 0.94 (0.76-1.17) | 0.575 |
| 2020 | 20,293 | 0.79 (0.63-0.98) | **0.029** |

† Negative binomial regression, using data by Hong Kong Hospital Authority cluster (Hong Kong East, Hong Kong West, Kowloon Central, Kowloon East, Kowloon West, New Territories East, New Territories West).
